# Supplementary material for: Structural Determinants of Substrate Specificity of Omega-3 Desaturases from Mortierella alpina and Rhizophagus irregularis by Domain-Swapping and Molecular Docking
Source: Int J Mol Sci. 2019 Mar 30;20(7):1603. doi: 10.3390/ijms20071603 (PMC6479736; doi:10.3390/ijms20071603)
Supplement: Supplementary file 1 [file ijms-20-01603-s001.pdf]

1. Supplemental Tables

Table S1. Primers used in this study

Primer name	Restriction enzyme	Oligo nucleotide sequence(5'-3') [a]	Function
**Fad-F**	*Eco*RI	CCGGAATTCATGGCTTCGTCCACCGTTG	FADS15 amplification for expression in *S. cerevisiae*
**Fad-R**	*Xho*I	CCGCTCGAGTTAGTTAGCCTTGGTCTTGGCAG	
**Rid-F**	*Eco*RI	CCGGAATTCATGTCGCCCTTGGAGC	oRiFADS17 amplification for expression in *S. cerevisiae*
**Rid-R**	*XhoI*	CCGCTCGAGTTACTGGTTCTTCTCCTTCTGG	
**T7**	-	TAATACGACTCACTATAGGG	Target genes detection for yeast transformants
**pYES2.R**	-	TCGGTTAGAGCGGATGTG	
**FA**	-	AAGTCCCTCCAGTACGTCGTCAAGGATCTGG	Overlap extension PCR for 12 fusion genes
**FB**	-	AAGTCGATCCTGCATGTCCTGTGGGACCTC	
**FC**	-	CACGACTGCGGCCACGGAGCGTTCTCGGAC	
**FD**	-	CACGAGTGCGGCCATGGTTCGTTCTCCCG	
**FE**	-	CACCGCCACCACCACAAGGGCACTGGATCC	
**FF**	-	CATTCCAAGCACCACAAGAACACCGGAAACATCG	
**FG**	-	GTTCCTGAAGAACCGTCGCAAGAACATTTTC	
**FH**	-	GTATGAGCCTCACCAGCTCGGTGCCATCATCTCG	
**FI**	-	GCTCGTGGCTGGTCATCATCACCTATCTCCAGC	
**FJ**	-	GCTTGGATCGTCTGCACCACCTTCCTCCACC	
**RA**	-	CCAGATCCTTGACGACGTACTGGAGGGACT T	
**RB**	-	GAGGTCCCACAGGACATGCAGGATCGACTT	
**RC**	-	GTCCGAGAACGCTCCGTGGCCGCAGTCGTG	
**RD**	-	CGGGAGAACGAACCATGGCCGCACTCGTG	
**RE**	-	GGATCCAGTGCCCTTGTGGTGGTGGCGGTG	

**RF**	-	CGATGTTTCCGGTGTTCTTGTGGTGCTTGGAATG	
**RG**	-	GAAAATGTTCTTGCGACGGTTCTTCAGGAAC	
**RH**	-	CGAGATGATGGCACCGAGCTGGTGAGGCTCATAC	
**RI**	-	GCTGGAGATAGGTGATGATGACCAGCCACGAGC	
**RJ**	-	GGTGGAGGAAGGTGGTGCAGACGATCCAAGC	
**W129T**		CACCATCTTTGGAACGGTCCTTCACTCTGC	Targetgeted mutagenesis for constructing FADS15 mutants
**V137T**		CACTCTGCTCTTTTGACGCCCTACCAGGCTTG	
**Y139F**		GCTCTTTTGGTGCCCTTCCAGGCTTGGGCC	
**S145T**		GGCTTGGGCCATGACGCATTCCAAGCACCAC	
**T144W**		CGACATCATCGGCTGGTTGCTGCACACCTTC	Targetgeted mutagenesis for constructing oRiFADS17 mutants
**V152T**		CACCTTCATCTTGACCCCCTACACCACCTGG	
**Y154F**		CATCTTGGTCCCCTTCACCACCTGGAAGCTG	
**S160T**		CCACCTGGAAGCTGACCCACCGCCACCACCAC	

[a] Underlined sequences indicate the additional restriction sites, fragments in FADS15 sequence or the mutant sites.

Table S2. Literature summary of catalytic efficiency of ω-3 desaturases from various species with LA, GLA, DGLA and AA substrates.

Rank [a]	Preference Index [b]	Strain name	The catalytic efficiency of ω3Des on LA (%) [c,d]	The catalytic efficiency of ω3Des on GLA (%)	The catalytic efficiency of ω3Des on DGLA (%)	The catalytic efficiency of ω3Des on AA (%)	Locus	Reference or source
+9	>>56	*Pythium aphanidermatum*	-	5.97	28.85	56.46	FW362186.1	15
+8	>>49	*Phytophthora sojae*	-	6.18	35.45	48.79	FW362213.1	15
+7	>>37	*Phytophthora ramorum*	-	4.70	31.02	37.12	FW362214.1	15
+6	>>31	*Phytophthora infestans*	-	-	-	30.94	CAJ30870.1	14
+5	>>26	*Saprolegnia diclina*	-	-	4.98	25.9	AY373823	13
+4	5	*Phytophthora parasitica*	7.11	5.63	25.34	49.70	KT372001	16
+3	5.1	*Octopus bimaculoides*	4.4	4.3	23.5	22.6	MH028785	Our lab
+2	5.1	*Caenorhabditis elegans*	11	-	0	≈ 56	CELE_Y67H2A.8	34
+1	1.7	*Rhizophagus irregularis*	34.2	41.8	61.8	58.5	MH028784	This study
0	1	*Pichia pastoris*	36.5	33.8	35.1	35.3	EF116884	33
-1	-1.7	*Mortierella alpina* 1S-4	11.50	8.90	3.60	6.70	AB182163	11
-2	-2.6	*Mortierella alpina* ATCC32222	62.37	63.99	41.82	23.67	AGZ84120.1	This study
-3	-4	*Magnaporthe grisea*	18.6	3.40	4.6	4.7	XP 362963	9
-4	-5	*Fusarium moniliforme*	49.30	15.70	17.50	9.80	DQ272516	9
-5	-6.2	*Fusarium graminearum*	17.40	3.10	5.80	2.80	EAA75859	9
-6	-13.8	*Saccharomyces kluyveri*	22.0	10.0	3.9	1.60	AB118663	7,8
-7	<<-10.6	*Perilla frutescens*	10.6	-	-	-	KX880389	12
-8	<<-16.9	*Salvia hispanica*	16.9	-	-	-	KX610653	12

[a] The "Rank" numbers represent the substrate preference level of corresponding ω3Des. E.g.: "+9" represents the strongest AA substrate preference; "-8" represents the strongest LA substrate preference; "0" represents no substrate preference.

[b] Preference indexes = [Catalytic efficiency EPA / Catalytic efficiency LA] or - [Catalytic efficiency LA/ Catalytic efficiency EPA].

[c] These data indicated catalytic efficiency of LA, GLA, DGLA and AA, respectively.

[d] The "-" represents catalytic efficiency not mentioned in corresponding reference, but its preference is known.

2. Supplemental Figures

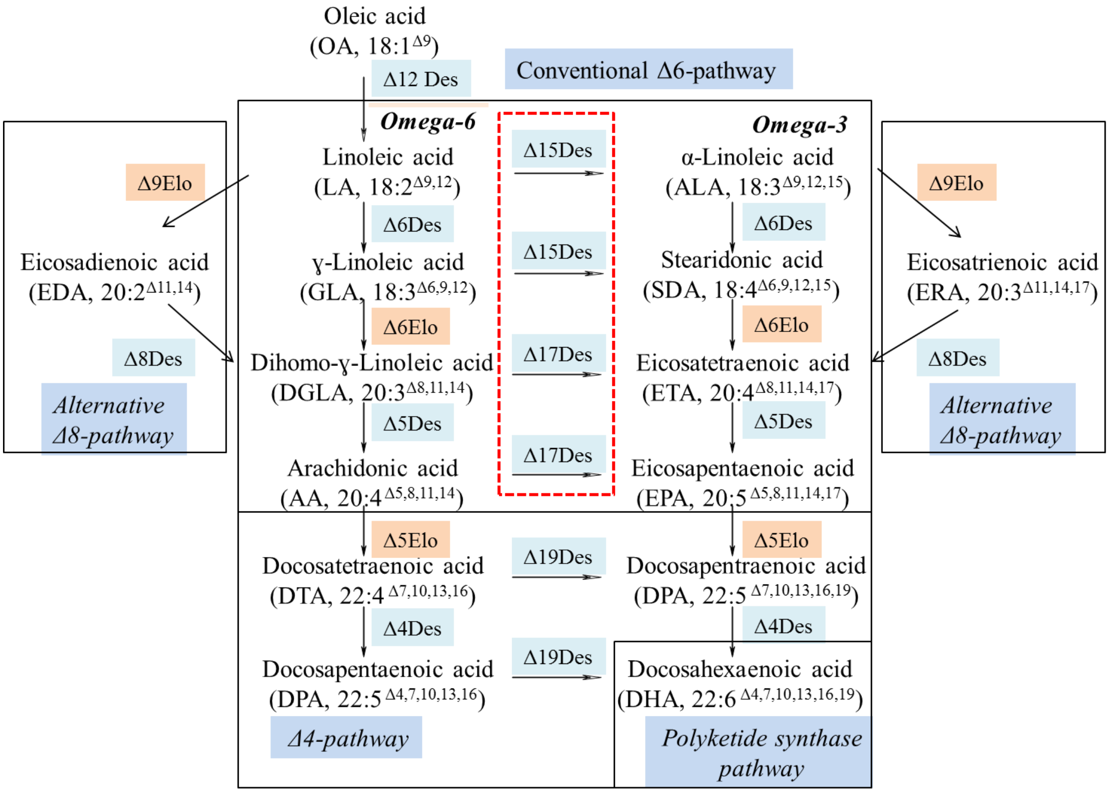

Figure S1. Schematic diagram of the fatty acid pathways for EPA and DHA synthesis.
